# A Genomics-Based Discovery of Secondary Metabolite Biosynthetic Gene Clusters in the Potential Novel Strain *Streptomyces* sp. 21So2-11 Isolated from Antarctic Soil

**DOI:** 10.3390/microorganisms12061228

**Published:** 2024-06-19

**Authors:** Yu Du, Wei Han, Puyu Hao, Yongqiang Hu, Ting Hu, Yinxin Zeng

**Affiliations:** 1 Key Laboratory for Polar Science, Polar Research Institute of China, Ministry of Natural Resources, Shanghai 200136, China; duyu@pric.org.cn (Y.D.); hanwei@pric.org.cn (W.H.); 18503887677@163.com (P.H.); huyongqiang@pric.org.cn (Y.H.); huting@pric.org.cn (T.H.); 2 College of Marine Ecology and Environment, Shanghai Ocean University, Shanghai 201306, China; 3 School of Oceanography, Shanghai Jiao Tong University, Shanghai 200030, China; 4 Antarctic Great Wall Ecology National Observation and Research Station, Polar Research Institute of China, Ministry of Natural Resources, Shanghai 200136, China; 5 Shanghai Key Laboratory of Polar Life and Environment Sciences, Shanghai Jiao Tong University, Shanghai 200030, China; 6 Key Laboratory of Polar Ecosystem and Climate Change, Shanghai Jiao Tong University, Ministry of Education, Shanghai 200030, China

**Keywords:** *Streptomyces*, genome mining, phylogenetic, biosynthetic gene cluster, secondary metabolite

## Abstract

*Streptomyces* species are attractive sources of secondary metabolites that serve as major sources of antibiotics and other drugs. In this study, genome mining was used to determine the biosynthetic potential of *Streptomyces* sp. 21So2-11 isolated from Antarctic soil. 16S rRNA gene sequencing revealed that this strain is most closely related to *Streptomyces drozdowiczii* NBRC 101007^T^, with a similarity of 98.02%. Genome comparisons based on average nucleotide identity (ANI) and digital DNA–DNA hybridization (dDDH) showed that strain 21So2-11 represents a novel species of the genus *Streptomyces*. In addition to a large number of genes related to environmental adaptation and ecological function, a total of 28 putative biosynthetic gene clusters (BGCs) responsible for the biosynthesis of known and/or novel secondary metabolites, including terpenes, lantipeptides, polyketides, nonribosomal peptides, RiPPs and siderophores, were detected in the genome of strain 21So2-11. In addition, a total of 1456 BGCs were predicted to contribute to the biosynthesis of more than 300 secondary metabolites based on the genomes of 47 *Streptomyces* strains originating from polar regions. The results indicate the potential of *Streptomyces* sp. 21So2-11 for bioactive secondary metabolite production and are helpful for understanding bacterial adaptability and ecological function in cold terrestrial environments.

## 1. Introduction

Actinomycetes are known for their potential to synthesize bioactive secondary metabolites (also known as natural products) that serve as major sources of agricultural and medical drugs, such as antibiotics and antifungal, antitumor, antiviral, antiparasitic, and immunosuppressive compounds [1,2], accounting for approximately 45% of the secondary metabolites of microbial origin [3]. The genus *Streptomyces* is considered the most effective producer of bioactive natural products widely used in clinical practice, such as streptomycin, kanamycin and vancomycin [4]. To date, more than 70% of currently known natural antibiotics from microorganisms are produced by *Streptomyces* species [5,6,7]. However, with the isolation of a large number of secondary metabolites, the probability of finding antibiotics with significant biological activity and novel structures from *Streptomyces* is decreased [8,9]. This has led to a shift in focus to unexplored, underexploited and/or extreme habitats for isolating novel actinobacteria, particularly novel *Streptomyces* species [10]. In addition, the development of new genetic tools and fermentation strategies is pursued to facilitate natural product discovery in the genus *Streptomyces* [11].

Due to their remote and isolated geographic locations and extreme and hostile environments, the Arctic and Antarctic regions are regarded as valuable places for the isolation of novel microorganisms. A large number of novel actinobacteria, including *Streptomyces* species, have been detected in Arctic and Antarctic environments [12,13,14,15]. In addition, to adapt to extreme conditions such as low temperature, polar days and strong ultraviolet radiation [16], microorganisms inhabiting Arctic and Antarctic environments have evolved unique gene regulations and metabolic functions and therefore have the potential to produce structurally novel secondary metabolites [17,18]. Secondary metabolites with unique structures and significant activities, including actinomycin V, cinerubin B [19], enterocin [20] and frigocyclinone [21], have been found in *Streptomyces* species isolated from Arctic and Antarctic habitats.

Recent advances in genome mining have promoted the discovery of natural products from microorganisms [22]. The genetic elements responsible for the biosynthesis of secondary metabolites are usually clustered in microbial genomes to form gene clusters. Genome mining has become an important approach to explore novel biosynthetic gene clusters (BGCs) from isolated strains or to isolate new bioactive microbial strains from the environment to find novel BGCs [23]. These novel BGCs have great potential for the production of novel natural products. In recent years, with the increase in the number of *Streptomyces* genomes published, *Streptomyces* genomes have been confirmed to contain a wide range of undiscovered BGCs, which can serve as an invaluable source for the discovery of novel drug leads [24]. Furthermore, a large number of novel BGCs have been discovered in the genomes of *Streptomyces* species originating from Arctic and Antarctic habitats [25,26,27], indicating that *Streptomyces* strains in polar regions have strong potential for containing new secondary metabolites. Therefore, identifying novel *Streptomyces* species in polar regions can provide a basis for mining novel genetic sources for new natural products.

In the present study, the bacterial strain 21So2-11 isolated from soil on the Fildes Peninsula, King George Island, Antarctica, was assigned to the genus *Streptomyces* and further identified as a novel species based on genomic analysis. Secondary metabolite BGCs in the genome of strain 21So2-11 were predicted and further compared with those of *Streptomyces* species originating from polar regions with genome data available in public databases to evaluate the potential for producing novel bioactive compounds found in *Streptomyces* species inhabiting Arctic and Antarctic environments.

## 2. Materials and Methods

### 2.1. Bacterial Isolation and Cultivation

Soil samples were collected from pristine sites and penguin-colony-impacted sites in the Fildes Peninsula region, King George Island, Antarctica, in December 2019. After removing surface 1 cm of the soil samples with a sterile spoon for decontamination, about 100 g of soil was collected in a sterile Whirl-Pak bag (Nasco, Fort Atkinson, WI, USA). The bacterial strain 21So2-11 was isolated from a pristine bulk soil sample (62°12′43.85″ S; 58°55′52.54″ W; approximately 28 m above sea level) collected on the top of a hill on Ardley Island. A measure of 1 g of the soil sample was suspended in 9 mL of sterile 0.85% (*w*/*v*) NaCl solution. The suspension was serially diluted to a 10^−5^ dilution, and 100 μL of the suspension was spread onto R2A plates (BD, Sparks, MD, USA). The plates were incubated at 15 °C for 30 days. Single colonies were purified by repeated streaking on R2A plates. Strain 21So2-11 was purified on R2A plates at 15 °C and preserved as a suspension in R2A broth containing glycerol (20%, *v*/*v*) at −80 °C. The strain was deposited at the Polar Research Institute of China under accession number PMCC100865. The growth range of temperature was tested in R2A broth at 4, 10, 15, 20, 25, 30, 35, 37 and 42 °C. Tolerance to NaCl was examined using R2A liquid media containing NaCl at concentrations ranging from 0 to 10% (*w*/*v*, in 1% intervals). In this study, strain 21So2-11 was routinely cultured in R2A broth or on R2A solidified with agar at 15 °C.

### 2.2. Genomic DNA Extraction, Whole Genome Sequencing and Genomic Analyses

The genomic DNA of the bacterium was extracted from liquid cultures using a Wizard^®^ Genomic DNA Purification Kit (Promega, Madison, WI, USA) according to the manufacturer’s protocol. The purified genomic DNA was subjected to sequencing on an Illumina NovaSeq platform by Shanghai Personalbio Technology Co., Ltd. (Shanghai, China). FastQC v0.11.7 was used to control the quality of the next-generation sequencing data using default parameters [28], and Trimmomatic v0.36 was used to remove adapters and low-quality reads using a 4 bp Q20 sliding window [29]. Filtered clean reads were assembled into scaffolds using SPAdes v3.12.0 [30]. Pilon v1.18 was utilized to correct the next-generation high-quality data and stitched together to assemble a complete genome sequence [31].

Coding DNA sequence (CDS) regions were predicted using GeneMarkS v4.32 with default settings [32]. tRNA and rRNA predictions were performed using tRNAscan-SE v1.3.1 [33] and Barrnap v0.9 (https://github.com/tseemann/barrnap; accessed on 11 February 2023), respectively. A graphical map of the circular genome was generated using online Proksee software (http://proksee.ca; accessed on 28 February 2024). All predicted protein-encoding genes were annotated using DIAMOND v0.8.36 BLASTP to perform sequence alignment based on the National Center for Biotechnology Information (NCBI) NR database, Clusters of Orthologous Groups (COG) database, and SwissProt database [34]. Gene annotation of KEGG (Kyoto Encyclopedia of Genes and Genomes) orthologs and pathways was completed using the KAAS v2.1 automated annotation system [35]. Genes were also annotated via Gene Ontology (GO) analysis using Blast2GO v1.0 [36]. Pathogenic and virulence-related genes in the genome were predicted by Virulence Factors of Pathogenic Bacteria (http://www.mgc.ac.cn/VFs/main.htm; accessed on 11 February 2023). Antibiotic resistance genes, antibiotic target genes, and antibiotic biosynthesis genes were predicted based on the Comprehensive Antibiotic Resistance Database (CARD, http://card.mcmaster.ca; accessed on 11 February 2023). Carbohydrate-active enzymes were predicted using the Carbohydrate-Active Enzymes Database (CAZy, http://www.cazy.org; accessed on 11 February 2023).

### 2.3. 16S rRNA Gene Sequencing and Phylogenetic Analysis

The 16S rRNA gene was amplified and sequenced as described previously [37]. Sequence similarity analysis of the almost-complete 16S rRNA gene sequence was performed using the GenBank database (http://blast.ncbi.nlm.nih.gov/Blast.cgi; accessed on 11 September 2022) and the EzBioCloud server (https://www.ezbiocloud.net; accessed on 11 September 2022). The 16S rRNA gene sequences of closely related type strains were downloaded from the GenBank database. Clustering and phylogenetic tree construction were performed using the maximum-likelihood (ML) algorithm based on the Kimura 2-parameter model in MEGA v11 [38] after multiple alignment of the data via ClustalW [39]. Bootstrap values from 1000 replications were used to determine the confidence level of the branches.

### 2.4. Phylogenomic Analysis

The genomic sequence was uploaded to the Type Strain Genome Server (TYGS; https://tygs.dsmz.de; accessed on 11 February 2024) for in silico-based taxonomic analysis [40]. Based on the dDDH value showing high similarity to strain 21So2-11, closely related type strains were chosen for phylogenomic analysis. The pairwise comparison of strain 21So2-11 with closely related type strains of the genus *Streptomyces* was performed using Genome BLAST Distance Phylogeny (GBDP), and accurate intergenomic distances were inferred via the “trimming” algorithm and distance formula d0. The intergenomic distances were used to construct a balanced minimum evolutionary tree using FastME v2.1.6.1 with 100 pseudobootstrap replicates for branch support. The online server TYGS platform was also used to determine the digital DNA–DNA hybridization (dDDH) values of strain 21So2-11 and its close neighbors. The genomic average nucleotide identity (ANI) values between strain 21So2-11 and its close relatives were calculated using an online ANI Calculator (https://www.ezbiocloud.net/tools/ani; accessed on 11 February 2024).

### 2.5. Comparative Analysis of Secondary Metabolite Biosynthetic Gene Clusters

To evaluate the biosynthetic capacity of strain 21So2-11, secondary metabolite BGC was identified in genomic sequences using antiSMASH 7.0 (https://antismash.secondarymetabolites.org; accessed on 11 February 2024). Furthermore, such identification of secondary metabolite BGCs was conducted in *Streptomyces* strains isolated from Arctic or Antarctic environments with genome data available in public databases. The BGC distribution across all the *Streptomyces* genomes investigated in this study was represented with a heatmap using the online ImageGP server (https://www.ehbio.com/ImageGP; accessed on 15 March 2024). Those BGCs were then clustered into groups based on sequence similarity using BiG-SCAPE v1.1.2 and CORASON workflows [41] using default parameters, including singletons. The MIBiG database v2.1 [42] was used to analyze networks to assign BGCs producing known compounds. The generated networks were visualized using Cytoscape v3.8.2 [43]. Comparison of the naphthomycin- and gaudimycin-producing gene clusters between strain 21So2-11 and other actinobacteria was performed using the multigene BLAST tool [44].

## 3. Results

### 3.1. 16S rRNA Gene- and Genome-Based Phylogenies of Streptomyces sp. 21So2-11

**Figure 1:**
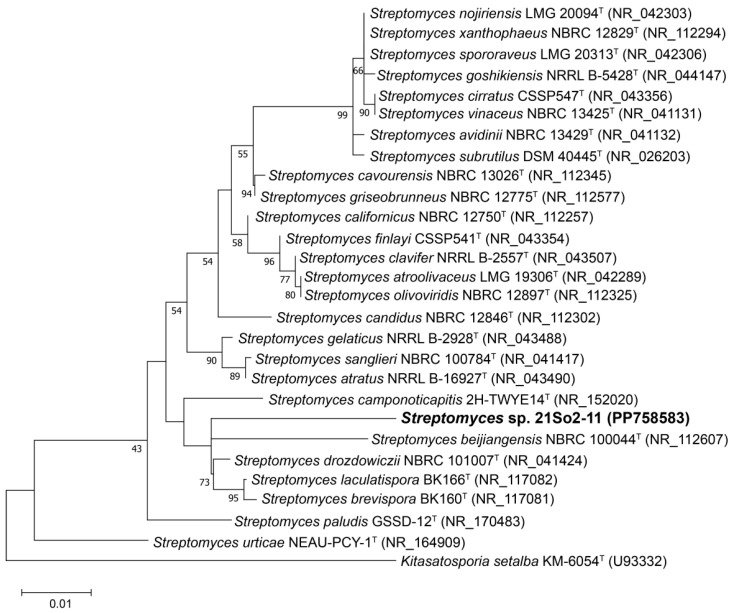
Maximum likelihood (ML) tree based on 16S rRNA gene sequences showing the phylogenetic position of strain 21So2-11 within the genus *Streptomyces*. Bootstrap values above 50% based on 1000 replicates are shown at branch nodes. *Kitasatospora setae* KM-6054^T^ was used as an outgroup. The scale bar corresponds to 0.01 substitutions per nucleotide position.

The 16S rRNA gene sequence of strain 21So2-11 (1486 bp) was aligned with the EzBioCloud database, which revealed that the strain shared the highest similarity with *Streptomyces drozdowiczii* NBRC 101007^T^ (98.02%), followed by *S. candidus* NBRC 12846^T^ (97.82%) and *S. avidinii* NBRC 13429^T^ (97.81%). The similarity values of strain 21So2-11 with the type strains of related *Streptomyces* species were below the recognized threshold (98.65%) for bacterial species definition [45], indicating that 21So2-11 represents a potential novel species of the genus *Streptomyces*. The 16S rRNA gene phylogenetic ML tree (Figure 1) revealed that strain 21So2-11 was clustered within the genus *Streptomyces* but formed a distinct line separated from the other *Streptomyces* species.

**Figure 2:**
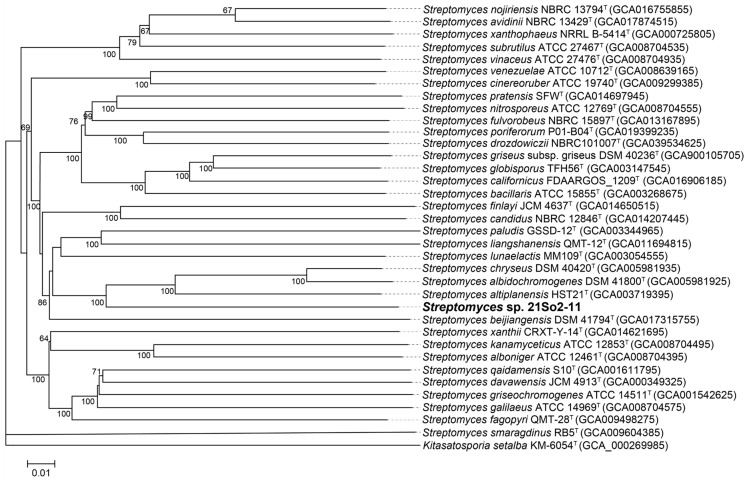
Whole-genome-sequence-based phylogenetic tree of strain 21So2-11 with closely related type strains. The numbers above the branches represent genome BLAST distance phylogeny (GBDP) pseudobootstrap values greater than 75% based on 100 replicates. The scale bar corresponds to 0.01 substitutions per nucleotide position. *Kitasatospora setae* KM-6054^T^ was used as an outgroup.

Compared to the 16S rRNA gene informing classification at and above the rank of genus, genome-based classification can afford greater resolution for delineations at the species and subspecies levels [46]. After the bacterial genome sequence was uploaded to TYGS, a total of 34 close *Streptomyces* reference strains were selected for phylogenetic analysis based on dDDH value showing high similarity to strain 21So2-11 (Table S1). The dDDH values between strain 21So2-11 and its most closely related species, *Streptomyces altiplanensis* HST21^T^, *S. chryseus* DSM 40420^T^ and *S. albidochromogenes* DSM 41800^T^, were 28.60%, 28.20% and 27.90%, respectively, which are well below the threshold value of 70% for species delineation [47]. Moreover, the dDDH values between strains 21So2-11 and *S. drozdowiczii* NBRC 101007^T^, *S. candidus* NBRC 12846^T^, and *S. avidinii* NBRC 13429^T^ were 23.30%, 23.30% and 22.70%, respectively. The genomic ANI values for strains 21So2-11 with *S. altiplanensis* HST21^T^, *S. chryseus* DSM 40420^T^ and *S. albidochromogenes* DSM 41800^T^ were 83.74%, 83.68% and 83.35%, respectively, which are lower than the cutoff value (95%) for species delineation [45]. The genomic ANI values between strains 21So2-11 and *S. drozdowiczii* NBRC 101007^T^, *S. candidus* NBRC 12846^T^, and *S. avidinii* NBRC 13429^T^ were 82.01%, 82.11% and 81.31%, respectively. The GBDP tree (Figure 2) showed that 21So2-11 was within the cluster composed of *S. altiplanensis* HST21^T^, *S. chryseus* DSM 40420^T^ and *S. albidochromogenes* DSM 41800^T^ but formed a distinct line separate from the three most closely related *Streptomyces* relatives. These results support that strain 21So2-11 represents a novel species of the *Streptomyces* genus, which is consistent with the results of 16S rRNA gene phylogenetic analysis.

### 3.2. Genomic Features of Streptomyces *sp.* 21So2-11

**Figure 3:**
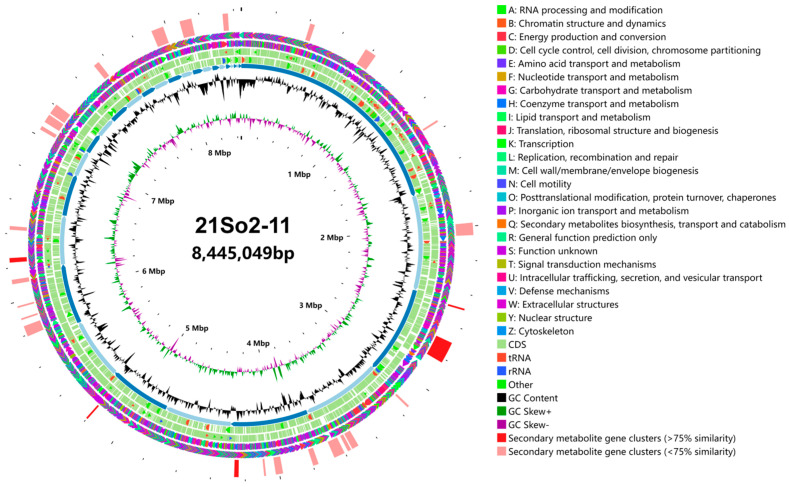
Circular visualization of the genome of *Streptomyces* sp. 21So2-11. The outer circle represents the distribution of gene clusters coding for secondary metabolites (red: clusters that are >75% similar to those BGCs present in related organisms; kermesinus: <75% similarity). The gene clusters are followed by COG on the forward (the second circle) and reverse (the third circle) strands (colored by COG categories). The fourth and fifth circles represent coding regions (CDSs), tRNAs (red bars) and rRNA operons (blue bars) in the sense and antisense directions, respectively. The order of the scaffolds is represented in the sixth circle. Histograms in the seventh circle indicate the GC content per 10,000 bases. The eighth circle represents GC skew data per 10,000 bases (green indicates positive skewness, and purple indicates negative skewness). The innermost circle represents the number of bases.

A total of 11,306,456 paired-end reads were obtained for *Streptomyces* sp. 21So2-11 after processing the high-quality reads. The coverage for the genome was approximately 198×. The genome had an N50 of 58.39 Kb and an L50 of 5. A circular map of *Streptomyces* sp. 21So2-11 is shown in Figure 3. After assembly, the draft genome of strain 21So2-11 had a total of 8,445,049 bp with 29 scaffolds. The DNA G + C content was estimated to be 69.57 mol%. A total of 7390 protein-encoding genes and 137 RNA genes (including 14 rRNA, 67 tRNA and 56 sRNA) were predicted in the genome. In addition, 6237 (84.39%), 4908 (66.41%) and 2445 (33.09%) genes were annotated by querying the COG, GO and KEGG databases, respectively. Among the 23 functional categories based on COG annotation, the 7 most abundant genes were those with functions related to unknown (category S; 34.41%), transcription (category K; 8.69%), replication, recombination and repair (category L; 8.21%), amino acid transport and metabolism (category E; 6.45%), carbohydrate transport and metabolism (category G; 5.98%), signal transduction mechanisms (category T; 5.13%), and energy production and conversion (category C; 4.73%) (Figure S1A).

### 3.3. Genes Related to Environmental Adaptation and Ecological Function

Cell growth of strain 21So2-11 was observed after incubation at 4–35 °C and 0–8.0% (*w*/*v*) NaCl, indicating its psychrotolerance and halotolerance. Genes related to environmental adaptation and ecological function were identified using KEGG and SwissProt annotations. A total of 36 genes related to salt and osmotic stress tolerance, including *betABIT*, *cvrA*, *dnaK*, *ectABCD*, *gbsA*, *gdh*, *gltBD*, *kdpABCDE*, *mscS*, *otsAB*, *osmC*, *prc*, *proABC*, *surE*, *treS* and *trkA*, were detected in the genome (Table S2). Furthermore, 154 genes related to membrane transport were detected based on KEGG annotation (Figure S1B). These genes may provide a protection strategy for strain 21So2-11 against salt and osmotic stress. In addition, a total of 22 cold-shock protein- and RNA helicase-related genes, including *cspAC*, *cstA*, *deaD*, *dinG*, *grpE*, *hepA*, *hmgA*, *hrpAB*, *recQ*, *rep*, *rhlE*, *rnr* and *tesB*, were detected in the genome of 21So2-11 (Table S2). Both cold-shock proteins and RNA helicases could play roles in the adaptation of the bacterial strain to the cold Antarctic environment. Moreover, a total of 21 genes responsible for repairing DNA damage induced by ultraviolet (UV) and ionizing radiation, including *lexA*, *mtcA*, *phr*, *polA*, *recAFORX*, *uvrABCD*, *rsr* and *ssb*, were detected in the genome of 21So2-11 (Table S2). Different gene families, different genes of the same gene family (e.g., *recAX* in scaffold5 and *recFOR* in scaffold1), and different copies of the same gene (e.g., four copies of *uvrD* in three different scaffolds) were found to be distributed at different positions in the genome (Table S2), indicating that the genes related to salt tolerance, cold adaptation and UV resistance were randomly distributed in the genome.

Strain 21So2-11 harbored 11 pathogenic and virulence-related genes, including *IdeR*, *DevR*, *MrpA*, *SigEH*, *GroEL* and *AhpC*. In addition, there were 33 antibiotic resistance genes, 21 antibiotic target genes and one antibiotic biosynthesis gene detected using the CARD database. A total of 291 putative CAZyme genes related to the degradation of polysaccharides, including 110 glycoside hydrolases, 68 glycosyl transferases, 57 carbohydrate esterases, 26 carbohydrate-binding modules, 22 auxiliary active enzymes and eight polysaccharide lyases, were found in the genome. These results suggest that strain 21So2-11 can compete with other bacteria and degrade polysaccharides for growth in the Antarctic terrestrial environment.

Based on KEGG annotation, complete key genes involved in the reductive citrate cycle (Arnon–Buchanan cycle), including *aclB*, *acnB*, *frdABCD*, *fumDE*, *korAB*, *mdh*, *ppc*, *ppdK*, *sdhABCD* and *sucCD*, were detected in the genome of strain 21So2-11, indicating its ecological role in carbon fixation. Moreover, the 21So2-11 genome contained all genes of the complete set of dissimilatory nitrate reduction pathways, including *narGHI* and *nirBD*. In addition, all genes of the complete set of assimilatory sulfate reduction pathways, including *cysCDHN* and *sir*, were detected in the bacterial genome. These results provide the genomic basis for the participation of strain 21So2-11 in carbon, nitrogen and sulfur metabolism in the local habitat.

### 3.4. Genome Mining of Secondary Metabolites

A total of 28 putative BGCs with an average length of 38,781 base pairs were identified, including 4 polyketide synthases (PKSs), 4 lantipeptides, 4 terpenes, 4 hybrid polyketide-nonribosomal peptides, 2 nonribosomal peptide synthetases (NRPSs), 2 siderophores, 2 RiPPs, 2 melanins, 1 ectoine, 1 LAP (linear azol(in)e-containing peptides), 1 hydrogen-cyanide, and 1 redox cofactor (Table S3). Among them, six gene clusters showed high similarity (>75%) to reported gene clusters responsible for melanin, coelichelin, isorenieratene, ectoine, SapB and hopene. For the other 22 BGCs, 6 of them shared similarities between 30 and 70% with known BGCs, whereas 12 BGCs likely encode novel compounds due to very low similarity values (<30%). The most four abundant types of secondary metabolite BGCs in the 21So2-11 genome were NRPSs, PKSs, NRPS-PKS hybrids, and terpenes. These BGCs included genes responsible for the synthesis of compounds with different biological activities, such as antitumor (aborycin), antimicrobial (auroramycin, capreomycin, enteromycin, gaudimycin, kinamycin, leucomycin, naphthomycin, neomediomycin and streptolydigin) and siderophore (desferrioxamine) activities.

**Figure 4:**
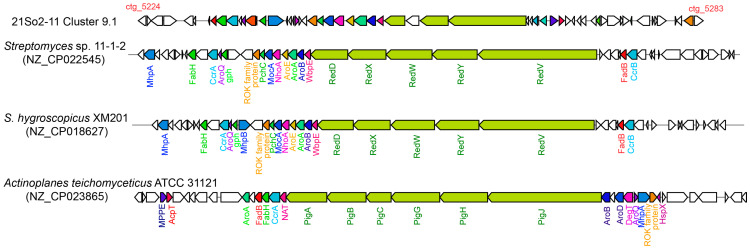
Comparison of the naphthomycin gene cluster in strain 21So2-11 (cluster 9.1 in Table S3) with those in *Streptomyces* sp. 11-1-2, *S. hygroscopicus* XM201 and *Actinoplanes teichomyceticus* ATCC 31121. Homologous genes among the four bacterial strains are shown in the same colors. Genes without any color in strain 21So2-11 are of unknown function, whereas those in the other species have no homologs in 21So2-11.

Two BGCs responsible for moldins (i.e., naphthomycin and gaudimycin) were predicted in strain 21So2-11. The bacterium had a hybrid polyketide-nonribosomal peptide system in cluster 9.1 (Table S3), which could be responsible for naphthomycin production. The naphthomycin biosynthesis gene cluster in strain 21So2-11 contained 60 genes, 27 of which were responsible for the production of the bioactive *red* pigment naphthomycin. The cluster comprised regulatory, transport-related, core, and additional genes involved in the biosynthesis of naphthomycin derivatives (Figure 4). The naphthomycin core biosynthesis gene cluster in strain 21So2-11 was identical to the *red* gene cluster found in *Streptomyces* sp. CS [48] and showed 95% similarity with that in *Streptomyces* sp. 11-1-2. The naphthomycin core synthesizing genes in *Streptomyces* sp. 11-1-2 and *S. hygroscopicus* XM201 were named the *red* gene cluster, whereas those in *Actinoplanes teichomyceticus* ATCC 31121 were identified as the *pig* gene cluster (Figure 4). In addition, strain 21So2-11 had a type II PKS system in cluster 19.1, which was responsible for gaudimycin production. This BGC exhibited 54% similarity to the gaudimycin C gene cluster found by Kallio et al. [49]. Gaudimycin core synthesizing genes in strains 21So2-11 and *Salinispora fenicalii* CNT-569 B116 were both identified as *fab* gene clusters (Figure S2). Additional biosynthetic genes responsible for SDR family oxidoreductases, the FMN reductase family and the TetR family, which are key enzymes in natural product biosynthetic pathways, were found in the 21So2-11 gaudimycin cluster (Figure S2). The presence of these genes suggests that *Streptomyces* sp. 21So2-11 has the potential to produce novel actinomycin analogs. In addition, another noteworthy BGC detected in strain 21So2-11 was the desferrioxamine gene cluster, which showed 50% similarity with the desferrioxamine B/E gene cluster in *S. coelicolor* [50]. Desferrioxamine can be used to treat iron overload disorders in humans [51].

### 3.5. Distribution of BGCs across Streptomyces Genomes Originating from Polar Regions

**Figure 5:**
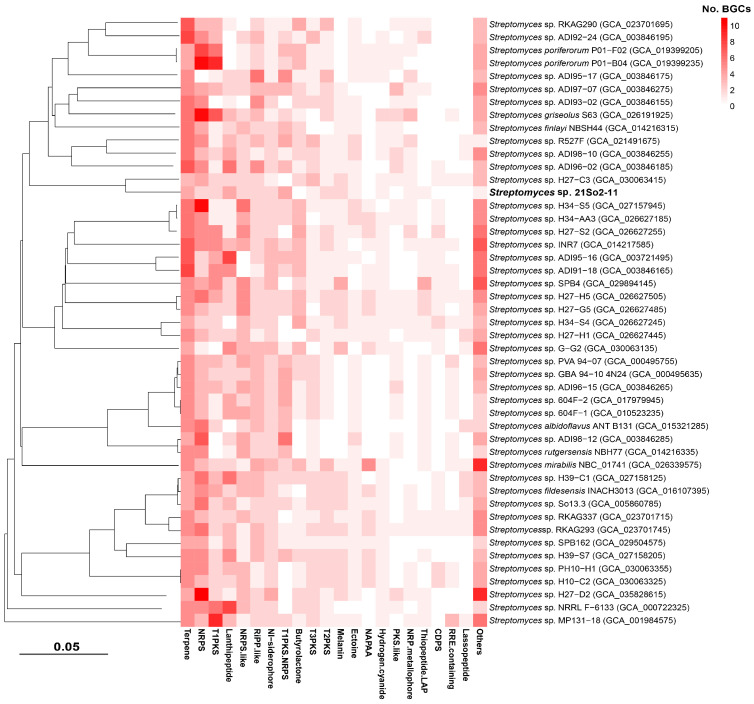
Heatmap of BGC types in the genomes of strain 21So2-11 and 46 other polar *Streptomyces* strains identified using antiSMASH and BiG-SCAPE.

**Figure 6:**
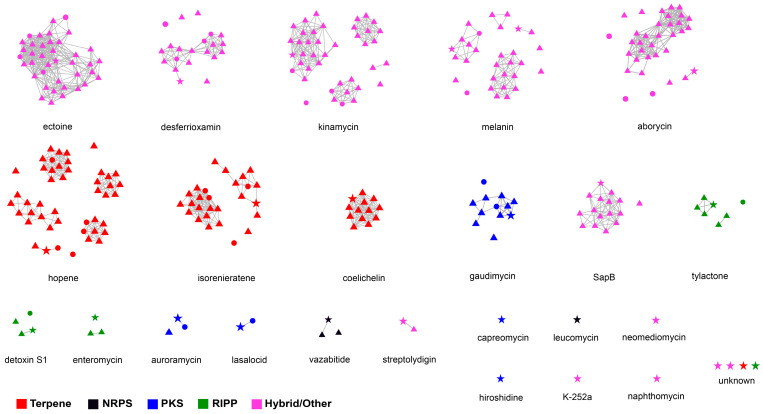
Sequence similarity network of 28 BGCs detected in strain 21So2-11 compared against BGCs in 46 other polar *Streptomyces* strains. Nodes in stars, triangles and circles represent BGCs originating from strain 21So2-11, 41 Antarctic *Streptomyces* strains, and 5 Arctic *Streptomyces* strains, respectively. Clusters of nodes associated with a MIBiG BGC are all presented separately. The colors are shown according to different BGC family annotations.

To evaluate the secondary metabolite biosynthetic potential of *Streptomyces* isolated from polar environments, BGCs from 47 selected *Streptomyces* genomes were identified using antiSMASH, and then a network analysis was performed in BiG-SCAPE together with similar BGC sequences available in the MIBiG database. A total of 1456 BGCs falling into 48 secondary metabolite categories were detected in the 47 polar *Streptomyces*, and the average number of BGCs in those genomes was 30.98. The four most abundant secondary metabolite categories were those related to terpenes (16.8% of total BGCs), NRPSs (15.2%), type I polyketide synthases (T1PKSs, 8.7%), and lanthipeptides (8.3%). Biosynthetic gene cluster families (BGCFs), including terpenes, NRPSs, T1PKSs, lanthipeptides and siderophores, were shared by almost all polar *Streptomyces* strains (Figure 5), which is consistent with previous studies [52]. In total, more than 300 secondary metabolites (including more than 100 antimicrobials) were found in the 47 polar *Streptomyces* strains, providing us with the opportunity to identify novel secondary metabolites in *Streptomyces* species inhabiting Arctic and Antarctic environments. The BGC types and related major secondary metabolites predicted on the basis of the genomes of the 47 investigated *Streptomyces* strains are shown in Figure S3. The network analysis revealed that among the 28 BGCs observed in strain 21So2-11, 24 BGCs showed 6–100% similarity to known BGCs according to the MIBiG database (Table S3), including 18 BGCs from *Streptomyces* strains originating from polar regions (Figure 6). These findings indicate that four novel BGCs in strain 21So2-11 have not been detected in reported actinomycetes and that ten BGCs in strain 21So2-11 have not been detected in known polar *Streptomyces* strains.

## 4. Discussion

In this study, both phylogenetic trees based on 16S rRNA gene sequences and whole-genome sequences (Figure 1 and Figure 2) revealed that strain 21So2-11 had a unique taxonomic position within the genus *Streptomyces*. Further genome comparisons based on ANI and dDDH confirmed that strain 21So2-11 is a potential novel *Streptomyces* species due to the fact that its similarity values with the closest type strains within the same genus were less than the thresholds for species definition. Thus, this actinomycete can likely be considered a new source for the discovery of novel secondary metabolites because of the well-known potential of *Streptomyces* in drug development [4,7].

A comprehensive evaluation of the genome of strain 21So2-11 revealed that the bacterium contained a large number of genes related to cold adaptation (e.g., cold-shock protein- and RNA helicase-related genes) and UV resistance (e.g., DNA damage repair-related genes) (Table S2), which are helpful for adapting to harsh Antarctic conditions such as low temperature and strong UV radiation [53,54]. The halotolerance (e.g., salt tolerance- and membrane transport-related genes) of strain 21So2-11, which was isolated from terrestrial soils, was an unexpected result. In fact, halotolerant *Streptomyces* strains have reportedly been isolated from terrestrial environments, including Antarctic soil [55,56]. A gene cluster responsible for ectoine synthesis was also detected in strain 21So2-11 (Table S3). As a main compatible solute, ectoine is beneficial for bacterial cells not only as an osmoregulatory solute but also as a protectant of cells by mitigating the detrimental effects of freezing, drying, UV radiation and cytotoxins [57]. Notably, most (83.0%) of the investigated *Streptomyces* strains isolated from various polar environments, including Arctic and Antarctic soils, Arctic and Antarctic sediments and Southern Ocean seawater, have been observed to contain ectoine and melanin-related BGCs with high similarity (Figure 5 and Figure 6), suggesting that the ectoine and melanin-related BGCs are core BGCs in polar *Streptomyces* species. In *Streptomyces* species, melanin is involved in virulence factors as well as in protection mechanisms against UV radiation and oxidative agents [58]. In addition, strain 21So2-11 contained a gene cluster responsible for synthesizing desferrioxamine, a siderophore that can be beneficial for the growth and development of *Streptomyces* [59]. The strain also contained a gene cluster responsible for the synthesis of SapB, a morphogenetic peptide important for the formation of aerial mycelia in *Streptomyces* [60]. These genes and gene clusters provide insight into the environmental adaptation and cell growth of *Streptomyces* sp. 21So2-11 inhabiting Antarctic soil.

Microorganisms have developed different strategies to prevent self-toxicity because they must be resistant to the biological effects of bioactive compounds to survive during natural product production/accumulation [61]. As the function of bacterial defense against self-toxicity is intimately linked to virulence factors and antibiotic resistance genes [62,63], abundant pathogenic and virulence-related genes as well as antibiotic resistance genes found in the 21So2-11 genome can contribute to preventing the self-toxicity of bacteria caused by secondary metabolites, including auroramycin, capreomycin, enteromycin, gaudimycin, leucomycin, naphthomycin, neomediomycin, streptolydigin and kinamycin. At the same time, these virulence factors and antibiotic resistance genes can provide strain 21So2-11 with an advantage in competing with organisms inhabiting the same habitat, especially those that are sensitive to the antibiotics produced by strain 21So2-11. In addition, four BGCs (i.e., Cluster Nos. 1.1, 4.3, 9.4 and 16.1; Table S3) in 21So2-11 showed no similarity to known gene clusters according to antiSMASH, indicating that these BGCs can contribute to the production of novel secondary metabolites by polar *Streptomyces* species.

Two BGCs related to naphthomycin and gaudimycin biosynthesis in strain 21So2-11 showed more than 50% similarity to known antibiotics. Naphthomycins are 29-membered naphthalenic ansamacrolactam antibiotics with antimicrobial and antineoplastic activities [48]. These antibiotics can act as fatty acid synthase inhibitors and have better inhibitory effects on both Gram-positive bacteria and fungi [64]. Naphthomycin also has antineoplastic activity by inhibiting various SH enzymes, particularly those involved in nucleic acid synthesis [65]. Angucyclines are a specific group of aromatic polyketides that are associated with various biological activities (e.g., antimicrobial and antitumor activities) and are mainly produced by soil-dwelling *Streptomyces* bacteria [66]. They can act as topoisomerase inhibitors by targeting bacterial and eukaryotic topoisomerase [67]. Through a cascade of oxidation/reduction reactions, the novel angucycline metabolite gaudimycin can be generated [49,68].

Abundant secondary metabolite BGCs (1456 BGCs) responsible for diverse known and/or novel secondary metabolites (48 secondary metabolite categories) were detected in the genomes of 47 investigated *Streptomyces* bacteria isolated from Arctic and Antarctic environments. These secondary metabolites, including ectoines, lanthipeptides, melanins, NRPSs, siderophores, terpenes, T1PKSs, T2PKSs and T3PKSs, have diverse chemical structures and various biological activities (e.g., antitumor and antibacterial activities), supporting their potential in drug development. Among the 1456 putative secondary metabolite BGCs, there were 162 unknown BGCs, accounting for 11.2% of the total BGCs. The four most abundant unknown BGCs were related to PKSs, NRPSs, terpenes and lanthipeptides, accounting for 19.1%, 18.0%, 14.8% and 8.6% of the total unknown BGCs, respectively. At the same time, high diversity was observed in the main putative secondary metabolites in the 47 polar *Streptomyces* strains based on genome mining. For example, the melanin family was divided into eight independent clades that have four groups and four individual types (Figure 6). In addition, the hopene family was composed of five groups and three individual types. Gene loss and horizontal gene transfer often lead to a highly variable distribution of BGCs in the *Streptomyces* genome, which allows them to lose or acquire BGCs in response to selective pressures [69]. Our results indicate that *Streptomyces* bacteria inhabiting polar regions are potentially valuable sources for identifying novel secondary metabolites.

Similarity network analysis clearly revealed that ten BGCs responsible for secondary metabolites, including capreomycin, hiroshidine, K-252a, leucomycin, naphthomycin, neomediomycin and four unknown materials, were exclusively detected in strain 21So2-11 (Figure 6), indicating the potential of this novel bacterial strain for the discovery of novel drug leads. Moreover, the results showed that this approach can complement antiSMASH analysis to provide a deeper insight into the biosynthetic potential of a given strain. Based on similarity network analysis, BGCs responsible for coelichelin, SapB, tylactone, enteromycin, vazabitide and streptolydigin were found in the Antarctic *Streptomyces* strains, whereas no secondary metabolite BGC was exclusively detected in the Arctic *Streptomyces* strains. Whether Antarctic actinomycetes can provide more chances to find known and/or novel natural products compared to Arctic bacteria should receive more attention in future research.

In the postgenome mining era, the continuously increasing number of *Streptomyces* genome sequences has provided invaluable genetic resources for the discovery of novel secondary metabolites with interesting biological activities [2]. However, most secondary metabolite BGCs in *Streptomyces* are silent or poorly expressed under laboratory culture conditions, limiting the effective use of *Streptomyces* bacteria [2,70]. Thus, the activation of silent secondary metabolite BGCs, including the expression of related functional genes and the regulation of biosynthetic pathways, is important for successfully isolating bioactive secondary metabolites from *Streptomyces* strains. Diverse methods have been applied to activate silent secondary metabolite BGCs, including culture media modifications, chemical or antibiotic treatments, co-cultivation and one strain many compounds method (OSMAC) [11,71]. However, these untargeted methods usually resulted in non-directed activation of silent secondary metabolite BGCs in *Streptomyces* [2]. Synthetic biology approaches including promoter replacement, overexpression or repression of regulatory genes, heterologous expression in different hosts and refactoring of targeted BGCs have been implemented for specific activation of target secondary metabolite BGCs. Over the last decade, synthetic biology has emerged as a powerful tool to facilitate natural product discovery via optimization of secondary metabolite production and activation of target silent BGCs in *Streptomyces* [2,11]. Synthetic biology strategies including engineering of original host genome and heterologous expression in chassis strains should be considered for expanding the productivity and diversity of available novel secondary metabolites from polar *Streptomyces* strains.

## 5. Conclusions

A draft genome was de novo assembled for strain 21So2-11, which was isolated from Antarctic soil, using Illumina sequencing technology. A combination of 16S rRNA gene-based phylogenetic analysis and genome-based identification revealed that strain 21So2-11 is a potential novel species within the genus *Streptomyces*. In addition to numerous genes related to environmental adaptation and ecological function, antiSMASH and sequence similarity network analyses revealed that the bacterium contained a large number of putative biosynthetic gene clusters responsible for known and/or novel secondary metabolites with various biological activities, such as antimicrobial and antitumor activities. Furthermore, the *Streptomyces* bacteria inhabiting the Antarctic may be valuable sources for identifying bioactive secondary metabolites. The results of this study provide important insights into the adaptation mechanism, ecological role and application potential of the novel *Streptomyces* sp. 21So2-11 inhabiting the Antarctic terrestrial environment.

## Data Availability

The GenBank accession numbers for the 16S rRNA gene sequence and the whole-genome shotgun project of *Streptomyces* sp. 21So2-11 are PP758583 and JBDHNH010000000 (BioProject: PRJNA1108015), respectively.

## Supplementary Materials

The following supporting information can be downloaded at https://www.mdpi.com/article/10.3390/microorganisms12061228/s1: Table S1: Genome characteristics of strain 21So2-11 and 34 closely related type strains used for phylogenetic analysis; Table S2: Genes related to cold adaptation, ultraviolet (UV) resistance and salt tolerance in the genome of *Streptomyces* sp. 21So2-11; Table S3: Putative secondary metabolite biosynthetic gene clusters (BGCs) in *Streptomyces* sp. 21So2-11 using the antiSMASH server (https://antismash.secondarymetabolites.org; accessed on 11 February 2024); Figure S1: Annotations of the genome of *Streptomyces* sp. 21So2-11 using the COG (A) and KEGG (B) databases; Figure S2. Comparison of the gaudimycin gene cluster in strain 21So2-11 (cluster 19.1 in Table S3) with that in *Salinispora fenicalii* CNT-569 B116. Homologous genes between the two bacterial strains are presented in the same colors and linked by brown lines. Genes without any color in strain 21So2-11 have unknown functions, whereas those in *S. fenicalii* CNT-569 B116 have no homologs in 21So2-11; Figure S3. BGC types and related major secondary metabolites (occurring more than once) in strain 21So2-11 and 46 other polar *Streptomyces* strains using antiSMASH and BiG-SCAPE.

## Abbreviations

ANI: Average Nucleotide Identity; BGCs, Biosynthetic Gene Clusters; BLAST, Basic Local Alignment Search Tool; CAZy, Carbohydrate-Active Enzymes Database; CDS, Coding Sequence; COG, Clusters of Orthologous Groups; dDDH, Digital DNA–DNA Hybridization; GBDP, Genome BLAST Distance Phylogeny; KEGG, Kyoto Encyclopedia of Genes and Genomes; NCBI, National Center for Biotechnology Information; NRPS, Non-Ribosomal Peptide Synthetase; PKS, PolyKetide Synthase; TYGS, Type Strain Genome Server.

## References

[1] Manivasagan P., Venkatesan J., Sivakumar K., Kim S.K. (2014). Pharmaceutically active secondary metabolites of marine actinobacteria. Microbiol. Res..

[2] Lee N., Hwang S., Lee Y., Cho S., Palsson B., Cho B.K. (2019). Synthetic biology tools for novel secondary metabolite discovery in *Streptomyces*. J. Microbiol. Biotechnol..

[3] Berdy J. (2005). Bioactive microbial metabolites. J. Antibiot..

[4] De Lima Procópio R.E., da Silva I.R., Martins M.K., de Azevedo J.L., de Araújo J.M. (2012). Antibiotics produced by *Streptomyces*. Braz. J. Infect. Dis..

[5] Takahashi Y., Nakashima T. (2018). Actinomycetes, an inexhaustible source of naturally occurring antibiotics. Antibiotics.

[6] Pham J.V., Yilma M.A., Feliz A., Majid M.T., Mafetone N., Walker J.R., Kim E., Cho H.J., Reynolds J.M., Song M.C. (2019). A review of the microbial production of bioactive natural products and biologics. Front. Microbiol..

[7] Sánchez-Suárez J., Coy-Barrera E., Villamil L., Díaz L. (2020). *Streptomyces*-derived metabolites with potential photo-protective properties-a systematic literature review and meta-analysis on the reported chemodiversity. Molecules.

[8] Newman D.J., Cragg G.M. (2020). Natural products as sources of new drugs over the nearly four decades from 01/1981 to 09/2019. J. Nat. Prod..

[9] Salwan R., Sharma V. (2020). Molecular and biotechnological aspects of secondary metabolites in actinobacteria. Microbiol. Res..

[10] Ates H., Saygin H., Cora M., Kilic A.O., Ay H. (2023). Genome-based classifcation of *Streptomyces anatolicus* sp. nov., an actinobacterium with antimicrobial and cytotoxic activities, and reclassifcation of *Streptomyces nashvillensis* as a later heterotypic synonym of *Streptomyces tanashiensis*. Antonie Leeuwenhoek.

[11] Krysenko S. (2023). Impact of nitrogen-containing compounds on secondary metabolism in *Streptomyces* spp.—A source of metabolic engineering strategies. SynBio.

[12] Le Roes-Hill M., Rohland J., Meyers P.R., Cowan D.A., Burton S.G. (2009). *Streptomyces hypolithicus* sp. nov., isolated from an Antarctic hypolith community. Int. J. Syst. Evol. Microbiol..

[13] Li J., Tian X.P., Zhu T.J., Yang L.L., Li W.J. (2011). *Streptomyces fildesensis* sp. nov., a novel streptomycete isolated from Antarctic soil. Antonie Leeuwenhoek.

[14] Zhang L., Ruan C., Peng F., Deng Z., Hong K. (2016). *Streptomyces arcticus* sp. nov., isolated from frozen soil. Int. J. Syst. Evol. Microbiol..

[15] Kamjam M., Nopnakorn P., Zhang L., Peng F., Deng Z., Hong K. (2019). *Streptomyces polaris* sp. nov. and *Streptomyces septentrionalis* sp. nov., isolated from frozen soil. Antonie Leeuwenhoek.

[16] Liu J.T., Lu X.L., Liu X.Y., Gao Y., Hu B., Jiao B.H., Zheng H. (2013). Bioactive natural products from the Antarctic and Arctic organisms. Mini. Rev. Med. Chem..

[17] Yukimura K., Nakai R., Kohshima S., Uetake J., Kanda H., Naganuma T. (2010). Spore-forming halophilic bacteria isolated from Arctic terrains: Implications for long-range transportation of microorganisms. Polar Sci..

[18] Tian Y., Li Y.L., Zhao F.C. (2017). Secondary metabolites from polar organisms. Mar. Drugs.

[19] Silva L.J., Crevelin E.J., Souza D.T., Lacerda-Júnior G.V., de Oliveira V.M., Ruiz L.T.G., Rosa L.H., Moraes L.A.B., Melo I.S. (2020). Actinobacteria from Antarctica as a source for anticancer discovery. Sci. Rep..

[20] Jiang S.P., Tian X.Q., Liao L., Yang Q., Lu Y.N., Ma L.Y., Chen B., Fan C.Q. (2016). Antimycin A and vulgamycin derivatives from the Arctic marine actinomycete *Streptomyces* sp. 604F. Chin. J. Polar Res..

[21] Bruntner C., Binder T., Pathom-aree W., Goodfellow M., Bull A.T., Potterat O., Puder C., Hörer S., Schmid A., Bolek W. (2005). Frigocyclinone, a novel angucyclinone antibiotic produced by a *Streptomyces griseus* strain from Antarctica. J. Antibiot..

[22] Schneider O., Simic N., Aachmann F.L., Rückert C., Kristiansen K.A., Jiang Y., Wang L.S., Jiang C.L., Lale R., Zotchev S.B. (2018). Genome mining of *Streptomyces* sp. YIM 130001 isolated from lichen affords new thiopeptide antibiotic. Front. Microbiol..

[23] Nguyen H.T., Pokhrel A.R., Nguyen C.T., Pham V.T.T., Dhakal D., Lim H.N., Jung H.J., Kim T.S., Yamaguchi T., Sohng J.K. (2020). *Streptomyces* sp. VN1, a producer of diverse metabolites including non-natural furan-type anticancer compound. Sci. Rep..

[24] Lee N., Kim W., Hwang S., Lee Y., Cho S., Palsson B., Cho B.K. (2020). Thirty complete *Streptomyces* genome sequences for mining novel secondary metabolite biosynthetic gene clusters. Sci. Data.

[25] Chen R.Q., Liao L., Zhang X.H., Chen B. (2014). Cloning and analysis of a halogenase gene of *Streptomyces* sp. 604F from the Arctic ocean. Acta. Microbiol. Sin..

[26] Guerrero-Garzón J.F., Zehl M., Schneider O., Rückert C., Busche T., Kalinowski J., Bredholt H., Zotchev S.B. (2020). *Streptomyces* spp. from the marine sponge *Antho dichotoma*: Analyses of secondary metabolite biosynthesis gene clusters and some of their products. Front. Microbiol..

[27] Duan Z.D., Liao L., Chen B. (2022). Complete genome analysis reveals secondary metabolite biosynthetic capabilities of *Streptomyces* sp. R527F isolated from the Arctic Ocean. Mar. Genom..

[28] Wingett S.W., Andrews S. (2018). FastQ Screen: A tool for multi-genome mapping and quality control. F1000Research.

[29] Bolger A.M., Lohse M., Usadel B. (2014). Trimmomatic: A flexible trimmer for Illumina sequence data. Bioinformatics.

[30] Bankevich A., Nurk S., Antipov D., Gurevich A.A., Dvorkin M., Kulikov A.S., Lesin V.M., Nikolenko S.I., Pham S., Prjibelski A.D. (2012). SPAdes: A new genome assembly algorithm and its applications to single-cell sequencing. J. Comput. Biol..

[31] Utturkar S.M., Klingeman D.M., Hurt R.A., Brown S.D. (2017). A case study into microbial genome assembly gap sequences and finishing strategies. Front. Microbiol..

[32] Besemer J., Lomsadze A., Borodovsky M. (2001). GeneMarkS: A self-training method for prediction of gene starts in microbial genomes. Implications for finding sequence motifs in regulatory regions. Nucleic Acids Res..

[33] Chan P.P., Lin B.Y., Mak A.J., Lowe T.M. (2021). tRNAscan-SE 2.0: Improved detection and functional classification of transfer RNA genes. Nucleic Acids Res..

[34] Buchfink B., Xie C., Huson D.H. (2015). Fast and sensitive protein alignment using DIAMOND. Nat. Methods.

[35] Moriya Y., Itoh M., Okuda S., Yoshizawa A.C., Kanehisa M. (2007). KAAS: An automatic genome annotation and pathway reconstruction server. Nucleic Acids Res..

[36] Conesa A., Götz S. (2008). Blast2GO: A comprehensive suite for functional analysis in plant genomics. Int. J. Plant Genom..

[37] Zeng Y.X., Zheng T.L., Li H.R. (2009). Community composition of the marine bacterioplankton in Kongsfjorden (Spitsbergen) as revealed by 16S rRNA gene analysis. Polar Biol..

[38] Tamura K., Stecher G., Kumar S. (2021). MEGA11: Molecular evolutionary genetics analysis version 11. Mol. Biol. Evol..

[39] Thompson J.D., Higgins D.G., Gibson T.J. (1994). CLUSTAL W: Improving the sensitivity of progressive multiple sequence alignment through sequence weighting, position-specific gap penalties and weight matrix choice. Nucleic Acids Res..

[40] Meier-Kolthof J.P., Göker M. (2019). TYGS is an automated high-throughput platform for state-of-the-art genome-based taxonomy. Nat. Commun..

[41] Blin K., Shaw S., Steinke K., Villebro R., Ziemert N., Lee S.Y., Medema M.H., Weber T. (2019). AntiSMASH 5.0: Updates to the secondary metabolite genome mining pipeline. Nucleic Acids Res..

[42] Kautsar S.A., Blin K., Shaw S., Navarro-Munoz J.C., Terlouw B.R., van der Hooft J.J.J., van Santen J.A., Tracanna V., Suarez Duran H.G., Pascal Andreu V. (2020). MIBiG 2.0: A repository for biosynthetic gene clusters of known function. Nucleic Acids Res..

[43] Shannon P., Markiel A., Ozier O., Baliga N.S., Wang J.T., Ramage D., Amin N., Schwikowski B., Ideker T. (2003). Cytoscape: A software environment for integrated models of biomolecular interaction networks. Genome Res..

[44] Medema M.H., Takano E., Breitling R. (2013). Detecting sequence homology at the gene cluster level with multigeneblast. Mol. Biol. Evol..

[45] Kim M., Oh H.S., Park S.C., Chun J. (2014). Towards a taxonomic coherence between average nucleotide identity and 16S rRNA gene sequence similarity for species demarcation of prokaryotes. Int. J. Syst. Evol. Microbiol..

[46] Hugenholtz P., Chuvochina M., Oren A., Parks D.H., Soo R.M. (2021). Prokaryotic taxonomy and nomenclature in the age of big sequence data. ISME J..

[47] Chun J., Oren A., Ventosa A., Christensen H., Arahal D.R., da Costa M.S., Rooney A.P., Yi H., Xu X.W., De Meyer S. (2018). Proposed minimal standards for the use of genome data for the taxonomy of prokaryotes. Int. J. Syst. Evol. Microbiol..

[48] Wu Y.Y., Kang Q.J., Shen Y.M., Su W.J., Bai L.Q. (2011). Cloning and functional analysis of the naphthomycin biosynthetic gene cluster in *Streptomyces* sp. CS. Mol. Biosyst..

[49] Kallio P., Liu Z.L., Mäntsälä P., Niemi J., Metsä-Ketelä M. (2008). Sequential action of two flavoenzymes, *PgaE* and *PgaM*, in angucycline biosynthesis: Chemoenzymatic synthesis of gaudimycin C. Cell Chem. Biol..

[50] Barona-Gómez F., Wong U., Giannakopulos A.E., Derrick P.J., Challis G.L. (2004). Identifcation of a cluster of genes that directs desferrioxamine biosynthesis in *Streptomyces coelicolor* M145. J. Am. Chem. Soc..

[51] Müller G., Raymond K.N. (1984). Specificity and mechanism of ferrioxamine-mediated iron transport in *Streptomyces pilosus*. J. Bacteriol..

[52] Park C.J., Andam C.P. (2019). Within-species genomic variation and variable patterns of recombination in the tetracycline producer *Streptomyces rimosus*. Front. Microbiol..

[53] Marizcurrena J.J., Herrera L.M., Costabile A., Morales D., Villadoniga C., Eizmendi A., Davyt D., Castro-Sowinski S. (2019). Validating biochemical features at the genome level in the Antarctic bacterium *Hymenobacter* sp. strain UV11. FEMS Microbiol. Lett..

[54] De Francisco Martínez P., Morgante V., González-Pastor J.E. (2022). Isolation of novel cold-tolerance genes from rhizosphere microorganisms of Antarctic plants by functional metagenomics. Front. Microbiol..

[55] Bhave S.V., Shanbhag P.V., Sonawane S.K., Parab R.R., Mahajan G.B. (2013). Isolation and characterization of halotolerant *Streptomyces radiopugnans* from Antarctica soil. Lett. Appl. Microbiol..

[56] Mohamed S.H., Al-Saeedi T.A., Sadik A.S. (2013). Halotolerant streptomycetes isolated from soil at Taif region, Kingdom of Saudi Arabia II: RAPD-PCR analysis and salt tolerance-gene isolation. Afr. J. Biotechnol..

[57] Schwibbert K., Marin-Sanguino A., Bagyan I., Heidrich G., Lentzen G., Seitz H., Rampp M., Schuster S.C., Klenk H.P., Pfeifer F. (2011). A blueprint of ectoine metabolism from the genome of the industrial producer *Halomonas elongata* DSM 2581^T^. Environ. Microbiol..

[58] Kordjazi T., Mariniello L., Giosafatto C.V.L., Porta R., Restaino O.F. (2024). Streptomycetes as microbial cell factories for the biotechnological production of melanin. Int. J. Mol. Sci..

[59] Codd R., Richardson-Sanchez T., Telfer T.J., Gotsbacher M.P. (2018). Advances in the chemical biology of desferrioxamine B. ACS Chem. Biol..

[60] Kodani S., Hudson M.E., Durrant M.C., Buttner M.J., Nodwell J.R., Willey J.M. (2004). The SapB morphogen is a lantibiotic-like peptide derived from the product of the developmental gene *ramS* in *Streptomyces coelicolor*. Proc. Natl. Acad. Sci. USA.

[61] Almabruk K.H., Dinh L.K., Philmus B. (2018). Self-resistance of natural product producers: Past, present, and future focusing on self-resistant protein variants. ACS Chem. Biol..

[62] Castillo-Arteaga R.D., Garrido L.M., Pedre B., Helmle I., Gross H., Gust B., Padilla G. (2022). Mycothiol peroxidase activity as a part of the self-resistance mechanisms against the antitumor antibiotic Cosmomycin, D. Microbiol. Spectr..

[63] Hu X., Tang Y., Liu Y., Pei X., Huang Z., Song F., Zhang H. (2022). Comprehensive genomic analysis of marine strain *Streptomyces* sp. 891, an excellent producer of Chrysomycin A with therapeutic potential. Mar. Drugs.

[64] Mukhopadhyay T., Franco C.M.M., Reddy G.C.S., Ganguli B.N., Fehlhaber H.W. (1985). A new ansamycin antibiotic, naphthomycin H from a *Streptomyces* species Y-83, 40369. J. Antibiot..

[65] Okabe T., Yuan B.D., Isono F., Sato I., Fukazawa H., Nishimura T., Tanaka N. (1985). Studies on antineoplastic activity of naphthomycin, a naphthalenic ansamycin, and its mode of action. J. Antibiot..

[66] Patrikainen P., Kallio P., Fan K., Klika K.D., Shaaban K.A., Mäntsälä P., Rohr J., Yang K., Niemi J., Metsä-Ketelä M. (2012). Tailoring enzymes involved in the biosynthesis of angucyclines contain latent context-dependent catalytic activities. Chem. Biol..

[67] Klahn P., Brönstrup M. (2017). Bifunctional antimicrobial conjugates and hybrid antimicrobials. Nat. Prod. Rep..

[68] Palmu K., Ishida K., Mäntsälä P., Hertweck C., Metsä-Ketelä M. (2007). Artificial reconstruction of two cryptic angucycline antibiotic biosynthetic pathways. ChemBioChem.

[69] Belknap K.C., Park C.J., Barth B.M., Andam C.P. (2020). Genome mining of biosynthetic and chemotherapeutic gene clusters in *Streptomyces* bacteria. Sci. Rep..

[70] Onaka H. (2017). Novel antibiotic screening methods to awaken silent or cryptic secondary metabolic pathways in actinomycetes. J. Antibiot..

[71] Lim F.Y., Sanchez J.F., Wang C.C., Keller N.P. (2012). Toward awakening cryptic secondary metabolite gene clusters in filamentous fungi. Methods Enzymol..

