# Supplementary material for: A Genomics-Based Discovery of Secondary Metabolite Biosynthetic Gene Clusters in the Potential Novel Strain Streptomyces sp. 21So2-11 Isolated from Antarctic Soil
Source: Microorganisms. 2024 Jun 19;12(6):1228. doi: 10.3390/microorganisms12061228 (PMC11205464; doi:10.3390/microorganisms12061228)
Supplement: Supplementary file 1 [file microorganisms-12-01228-s001.zip › microorganisms-3062838-supplementary.pdf]

Supplementary Material

A Genomics-Based Discovery of Secondary Metabolite Biosynthetic Gene Clusters in the Potential Novel Strain *Streptomyces* sp. 21So2-11 Isolated from Antarctic Soil

Yu Du [1], Wei Han [1], Puyu Hao [1,2], Yongqiang Hu [1], Ting Hu [1] and Yinxin Zeng [1,3,4,5,6,*]

[1] Key Laboratory for Polar Science, Polar Research Institute of China, Ministry of Natural Resources, Shanghai 200136, China; duyu@pric.org.cn (Y.D.); hanwei@pric.org.cn (W.H.); 18503887677@163.com (P.H.); huyongqiang@pric.org.cn (Y.H.); huting@pric.org.cn (T.H.)
[2] College of Marine Ecology and Environment, Shanghai Ocean University, Shanghai 201306, China
[3] School of Oceanography, Shanghai Jiao Tong University, Shanghai 200030, China
[4] Antarctic Great Wall Ecology National Observation and Research Station, Polar Research Institute of China, Ministry of Natural Resources, Shanghai 200136, China
[5] Shanghai Key Laboratory of Polar Life and Environment Sciences, Shanghai Jiao Tong University, Shanghai 200030, China
[6] Key Laboratory of Polar Ecosystem and Climate Change, Shanghai Jiao Tong University, Ministry of Education, Shanghai 200030, China
* Correspondence: zengyinxin@pric.org.cn; Tel.: +86-21-5871-7207

1 Supplementary Table

Table S1. Genome characteristics of strain 21So2-11 and 34 close related type strains used for phylogenetic analysis.

Strain	Genome size (Mb)	G+C content (%)	Contigs number	N50 (Kb)	CDS	dDDH (%)	ANI (%)
Streptomyces sp. 21So2-11	8.45	69.57	29	58.39	7,390	100.00	100.00
S. altiplanensis HST21^{T}	7.92	70.98	459	37.40	-	28.60	83.74
S. chryseus DSM 40420^{T}	7.08	71.35	1,451	9.50	6,500	28.20	83.68
S. albidochromogenes DSM 41800^{T}	7.27	71.47	795	18.70	6,526	27.90	83.35
S. lunaelactis MM109^{T}	8.57	69.75	1	8,570.00	7,366	24.70	83.00
S. poriferorum P01-B04^{T}	8.91	70.73	695	26.90	7,671	24.00	82.21
S. beijiangensis DSM 41794^{T}	8.40	69.63	1,769	21.70	7,802	23.80	82.10
S. bacillaris ATCC 15855^{T}	7.89	71.95	1	7,890.00	6,509	23.70	82.00
S. paludis GSSD-12^{T}	8.45	71.19	1	8,450.00	6,960	23.60	81.98
S. californicus FDAARGOS_1209^{T}	8.03	72.56	3	7,800.00	6,840	23.60	82.16
S. liangshanensis QMT-12^{T}	7.72	72.10	1	7,720.00	6,538	23.40	82.03
S. fulvorobeus NBRC 15897^{T}	6.63	70.80	2	6,600.00	5,321	23.40	82.03
S. griseus subsp. griseus DSM 40236^{T}	8.63	72.25	4	6,000.00	7,068	23.30	82.18
S. candidus NBRC 12846^{T}	7.40	71.15	64	349.20	6,430	23.30	82.11
S. drozdowiczii NBRC 101007^{T}	7.36	72.01	82	413.70	6,316	23.30	82.01
S. globisporus TFH56^{T}	7.67	71.54	3	7,500.00	6,492	23.30	82.08
S. nitrosporeus ATCC 12769^{T}	7.58	72.17	1	7,580.00	6,461	23.20	81.78
S. finlayi JCM 4637^{T}	9.49	71.32	65	413.30	8,177	23.10	81.88
S. cinereoruber ATCC 19740^{T}	7.52	72.89	1	7,520.00	6,545	23.10	81.74

S. pratensis SFW^{T}	7.71	70.94	8	6,200.00	4,667	23.10	81.87
S. nojiriensis NBRC 13794^{T}	9.05	71.98	6	5,900.00	7,972	22.90	81.37
S. venezuelae ATCC 10712^{T}	8.22	72.46	1	8,220.00	7,265	22.90	81.75
S. vinaceus ATCC 27476^{T}	7.67	72.34	1	7,670.00	6,735	22.80	81.42
S. avidinii NBRC 13429^{T}	8.36	71.85	68	567.40	7,316	22.70	81.31
S. subrutilus ATCC 27467^{T}	7.60	73.45	1	7,600.00	6,555	22.70	81.43
S. kanamyceticus ATCC 12853^{T}	10.13	71.03	1	10,130.00	8,529	22.70	81.41
S. alboniger ATCC 12461^{T}	7.96	71.16	1	7,960.00	6,741	22.60	81.39
S. xanthophaeus NRRL B-5414^{T}	8.62	72.24	163	253.50	7,667	22.50	81.21
S. fagopyri QMT-28^{T}	8.88	71.24	1	8,880.00	7,429	22.50	81.15
S. xanthii CRXT-Y-14^{T}	8.35	72.15	3	7,900.00	7,442	22.00	80.85
S. galilaeus ATCC 14969^{T}	7.76	71.44	1	7,760.00	6,808	22.00	80.78
S. griseochromogenes ATCC 14511^{T}	10.76	70.76	1	10,760.00	9,277	22.00	80.75
S. qaidamensis S10^{T}	9.08	71.26	1	9,080.00	7,871	21.80	80.54
S. davawensis JCM 4913^{T}	9.56	70.58	2	9,500.00	8,396	21.80	80.47
S. smaragdinus RB5^{T}	7.86	72.02	106	161.60	6,985	21.30	79.71

Table S2. Genes related to cold adaptation, ultraviolet (UV) resistance and salt tolerance in the genome of *Streptomyces* sp. 21So2-11.

Type	Database	Scaffold No.	Start-End	Gene name
Cold-shock protein- and RNA helicase	KEGG, SwissProt	Scaffold1, 3, 4, 10, 14, 18	Scaffold1 (324,347-324,550); Scaffold3 (188,521-188,724); Scaffold4 (334,330-334,533); Scaffold10 (189,984-190,172); Scaffold14 (93,119-93,322); Scaffold18 (96,992-97,198)	*cspA* (6 copies)
	SwissProt	Scaffold1	294,634-295,017	cspC
	KEGG, SwissProt	Scaffold2	465,520-467,667	cstA
	KEGG	Scaffold11	114,067-116,622	deaD
	KEGG, SwissProt	Scaffold5	65,828-67,798	dinG
	SwissProt	Scaffold1	534,859-535,530	grpE
	KEGG	Scaffold7	287,815-290,625	hepA
	SwissProt	Scaffold6	400,848-402,167	hmgA
	KEGG, SwissProt	Scaffold1	1,051,730-1,055,620	hrpA
	KEGG, SwissProt	Scaffold7	242,433-245,009	hrpB
	KEGG, SwissProt	Scaffold5, 8	Scaffold5 (81,954-84,125); Scaffold8 (415,861-417,957)	*recQ* (2 copies)
	SwissProt	Scaffold17	106,956-108,419	rep
	KEGG, SwissProt	Scaffold1	1,062,618-1,064,969	rhlE
	KEGG, SwissProt	Scaffold14	26,486-27,925	rnr
	KEGG, SwissProt	Scaffold1, 9	1,379,979-1,380,851; 199,270-200,151	*tesB* (2 copies)
Repair of DNA damage induced by ultraviolet and	KEGG, SwissProt	Scaffold5	67,906-68,454	lexA
	SwissProt	Scaffold4	67,576-68,256	mtcA
	KEGG, SwissProt	Scaffold6	266,256-267,617	phr
	KEGG, SwissProt	Scaffold7	235,422-238,079	polA

ionizing radiation	KEGG, SwissProt	Scaffold5	30,736-31,902; 31,918-32,499	recA; recX
	KEGG, SwissProt	Scaffold1	839,527-840,657; 1,660,666-1,661,421; 481,861-482,460	recF; recO; recR
	SwissProt	Scaffold2	477,956-478,279	rsr
	KEGG, SwissProt	Scaffold1	876,748-877,380	ssb
	SwissProt	Scaffold1, 4, 7, 12	Scaffold1 (1,005,377-1,007,662); Scaffold4 (430,674-433,082); Scaffold7 (317,406-320,408); Scaffold12 (18,549-21,563)	*uvrA* (4 copies)
	KEGG, SwissProt	Scaffold7	309,409-311,538; 321,917-324,004	uvrB; uvrC
	KEGG, SwissProt	Scaffold1, 2, 8	Scaffold1 (304,110-306,194); Scaffold2 (512,499-514,643); Scaffold2 (517,708-521,637); Scaffold8 (242,609-245,110)	*uvrD* (4 copies)
Salt and osmotic stress tolerance	KEGG	Scaffold3	151,729-153,300; 148,632-150,101	betA; betB
	KEGG, SwissProt	Scaffold10	39,111-39,707; 80,783-82,528	betI; betT
	KEGG	Scaffold17	95,111-96,637	cvrA
	KEGG, SwissProt	Scaffold1	535,527-537,404	dnaK
	SwissProt	Scaffold7	399,546-400,034; 397,795-398,193; 396,872-397,789	ectA; ectC; ectD
	KEGG	Scaffold5, 7	Scaffold5 (59,644-61,023); Scaffold7 (398,251-399,519)	*ectB* (2 copies)
	KEGG, SwissProt	Scaffold8	203,188-204,690	gbsA
	KEGG, SwissProt	Scaffold1, 10	Scaffold1 (1,686,327-1,687,097); Scaffold10 (37,184-38,362)	*gdh* (2 copies)
	KEGG, SwissProt	Scaffold7, 21	Scaffold7 (175,646-180,265); Scaffold21 (49,449-50,735)	*gltB* (2 copies)
	KEGG, SwissProt	Scaffold7	180,399-181,718	gltD
	KEGG, SwissProt	Scaffold5	240,438-242,102; 244,206-244,892; 245,113-247,656; 247,727-248,410	kdpA; kdpC; kdpD; kdpE
	KEGG, SwissProt	Scaffold5	242,099-244,201; 548,650-550,686	*kdpB* (2 copies)
	SwissProt	Scaffold1	1,494,450-1,495,508	mscS

KEGG, SwissProt	Scaffold1	327,933-329,354; 329,656-330,510	otsA; otsB
KEGG, SwissProt	Scaffold4, 5, 12	Scaffold4 (293,259-293,684); Scaffold5 (419,744-420,169); Scaffold12 (70,375-70,803)	*osmC* (3 copies)
KEGG, SwissProt	Scaffold1	1,169,400-1,170,575	prc
KEGG, SwissProt	Scaffold1	1,602,626-1,603,918; 1,600,710-1,601,843	proA; proB
KEGG, SwissProt	Scaffold11	311,918-312,727	proC
KEGG, SwissProt	Scaffold3	89,599-90,504	surE
KEGG, SwissProt	Scaffold2	227,899-229,620	treS
KEGG, SwissProt	Scaffold5	249,731-250,408	trkA

Table S3. Putative secondary metabolite biosynthetic gene clusters (BGCs) in *Streptomyces* sp. 21So2-11 using the antiSMASH server (https://antismash.secondarymetabolites.org).

Cluster No.	Location	Type	Most similar known cluster (%)
1.1	Scaffold1 (405,264-433,511)	Lanthipeptide, CDPS	-
1.2	Scaffold1 (760,173-829,116)	NRPS	Vazabitide A (30%)
1.3	Scaffold1 (1,346,427-1,358,139)	Siderophore	Desferrioxamine B/E (50%)
2.1	Scaffold2 (238,400-310,876)	T2PKS, oligosaccharide	Hiroshidine (41%)
3.1	Scaffold3 (30,843-41,304)	Melanin	Melanin (100%)
3.2	Scaffold3 (242,197-370,624)	Terpene	Coelichelin (100%)
4.1	Scaffold4 (17,674-30,557)	Hydrogen-cyanide	Aborycin (14%)
4.2	Scaffold4 (390,779-431,387)	T3PKS	Capreomycin IA/IB/IIA/IIB (12%)
4.3	Scaffold4 (439,466-460,572)	Terpene	-
4.4	Scaffold4 (482,190-546,826)	T1PKS, NRPS-like	Lasalocid (11%)
5.1	Scaffold5 (47,052-79,771)	Siderophore	Kinamycin (16%)
5.2	Scaffold5 (259,617-302,430)	NRPS-like	Leucomycin (11%)
5.3	Scaffold5 (359,191-370,636)	RiPP-like	Tylactone (6%)
5.4	Scaffold5 (519,082-544,975)	Terpene	Isorenieratene (100%)
7.1	Scaffold7 (392,795-403,193)	Ectoine	Ectoine (100%)
9.1	Scaffold9 (54,588-117,493)	T1PKS, NRPS-like	Naphthomycin A (53%)
9.2	Scaffold9 (155,028-165,372)	Melanin	Melanin (28%)

9.3	Scaffold9 (228,879-239,109)	RiPP-like	Detoxin S1 (44%)
9.4	Scaffold9 (369,202-395,652)	Lanthipeptide	-
10.1	Scaffold10 (42,334-68,907)	Terpene	Hopene (84%)
10.2	Scaffold10 (233,161-255,183)	Redox-cofactor	K-252a (14%)
12.1	Scaffold12 (161,433-184,027)	Lanthipeptide	SapB (100%)
13.1	Scaffold13 (18,562-68,579)	T1PKS	Auroramycin (17%)
13.2	Scaffold13 (77,896-140,453)	T1PKS, NRPS	Neomediomycin B (28%)
16.1	Scaffold16 (1,657-36,499)	Thiopeptide, LAP	-
19.1	Scaffold19 (25,480-97,962)	T2PKS, butyrolactone	Gaudimycin A/C/D (54%)
21.1	Scaffold21 (3,367-73,812)	Lanthipeptide, NRPS, T1PKS, blactam	Streptolydigin (13%)
25.1	Scaffold25 (5,829-37,472)	T1PKS, PKS-like	Enteromycin (20%)

2 Supplementary Figures

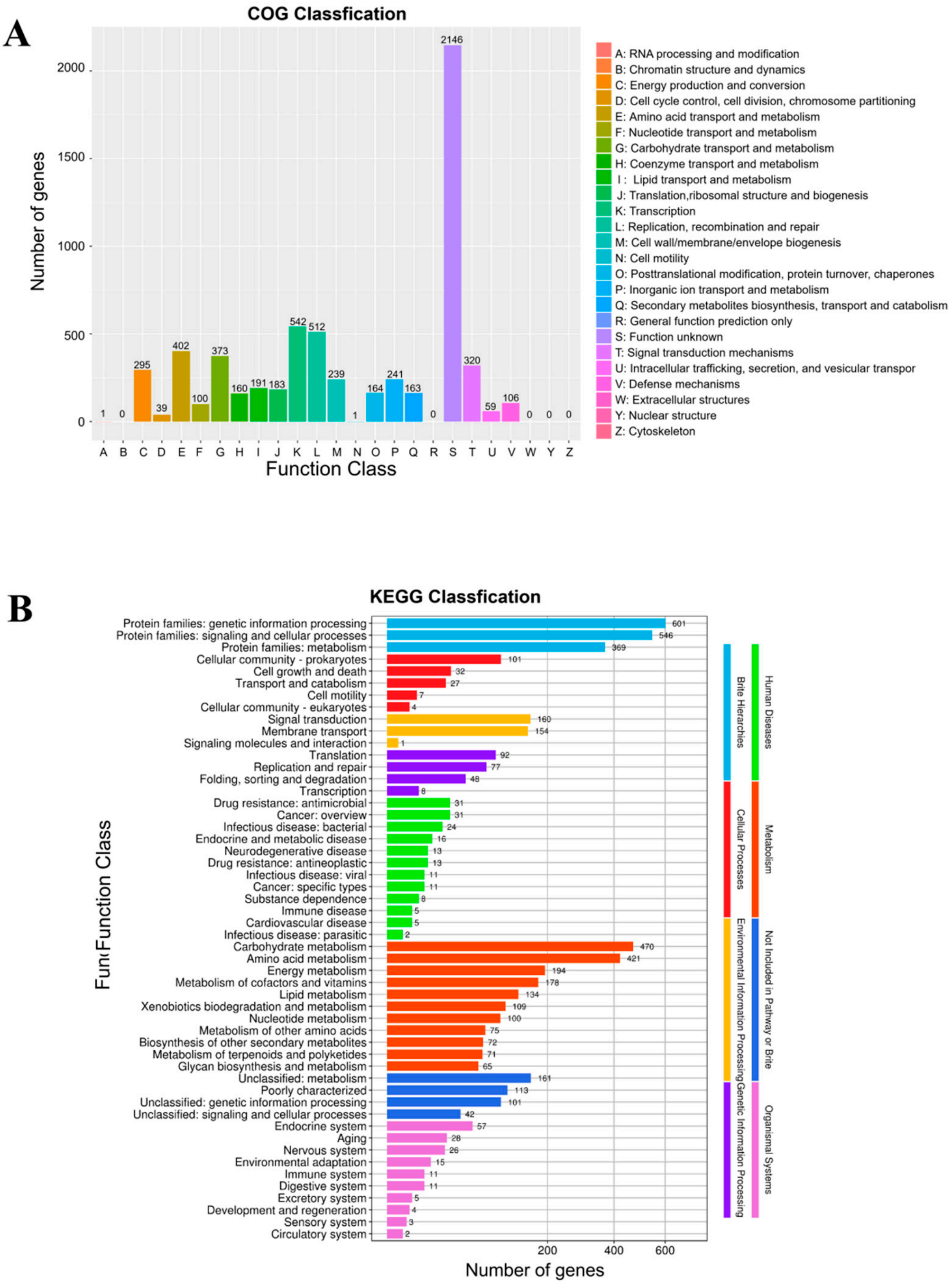

Figure S1. Annotations of the genome of *Streptomyces* sp. 21So2-11 using the COG (A) and KEGG (B) databases.

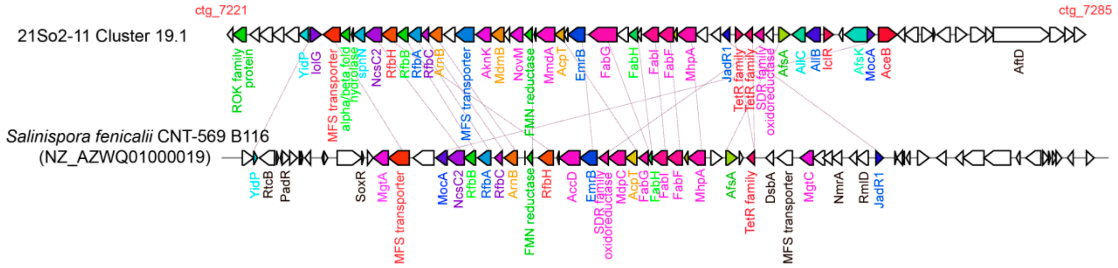

Figure S2. Comparison of the gaudimycin gene cluster in strain 21So2-11 (cluster 19.1 in **Table S3**) with that in *Salinispora fenicalii* CNT-569 B116. Homologous genes between the two bacterial strains are presented in the same colors and linked by brown lines. Genes without any color in strain 21So2-11 have unknown functions, whereas those in *S. fenicalii* CNT-569 B116 have no homologs in 21So2-11.

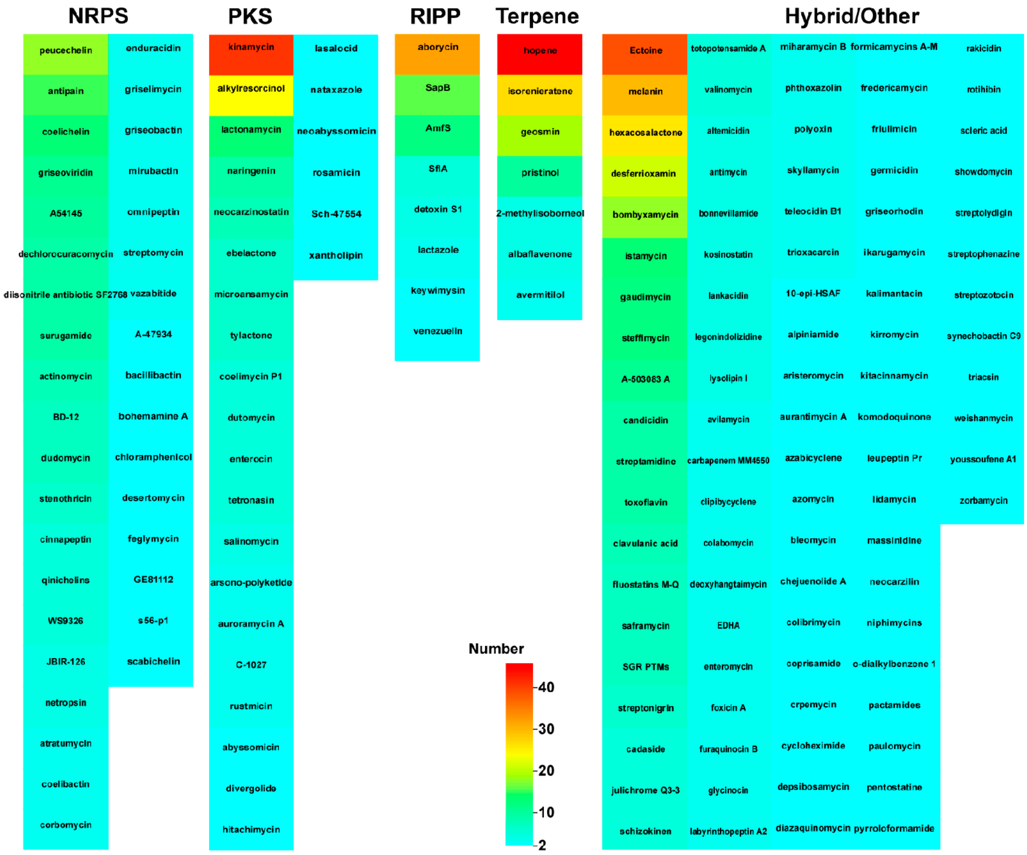

Figure S3. BGC types and related major secondary metabolites (occurring more than once) in strain 21So2-11 and 46 other polar *Streptomyces* strains using antiSMASH and BiG-SCAPE.
